# One Rhodopsin per Photoreceptor: Iro-C Genes Break the Rule

**DOI:** 10.1371/journal.pbio.0060115

**Published:** 2008-04-29

**Authors:** Doekele G Stavenga, Kentaro Arikawa

## Abstract

While photoreceptors usually contain a single type of rhodopsin, two rhodopsins are sometimes expressed. This bi-allelic expression appears to be under genetic control, an example of which is discussed in this Primer.

Ever since Morgan observed a mutant male with white eyes among the normally brilliant red-eyed specimens in his pedigree culture of fruit flies, Drosophila has been an inexhaustible source of scientific inspiration and discovery [1]. The regular, almost crystalline mosaic of the fruit fly eye has proved a powerful tool for unraveling genetic defects resulting in anatomical as well as functional modifications of normal physiological and/or behavioral patterns [2]. Developmental and cell biological studies in particular have greatly benefited from the clear and accessible organization of the fruit fly compound eye.

A Drosophila eye consists of roughly 800 anatomically identical units, called the ommatidia (Figure 1A). Each ommatidium contains eight photoreceptor cells (R1–R8) and is capped by a facet lens. The lens projects incident light into each photoreceptor's rhabdomere—the specialized, light-sensitive organelle that functions as a light guide and contains the visual pigment molecules (called rhodopsins). Rhabdomeres also harbor the photoreceptor's phototransduction machinery [3,4], which is made up of signaling components that capture and transform photons from light into an electrical signal, which conveys visual information to the brain. (All the rhabdomeres in an ommatidium are collectively known as the rhabdom.) The R1–R6 rhabdomeres stretch the full length of their photoreceptors, forming a trapezoid with the tandem of the R7 and R8 rhabdomeres in the center (Figure 1B and 1C).

**Figure 1:**
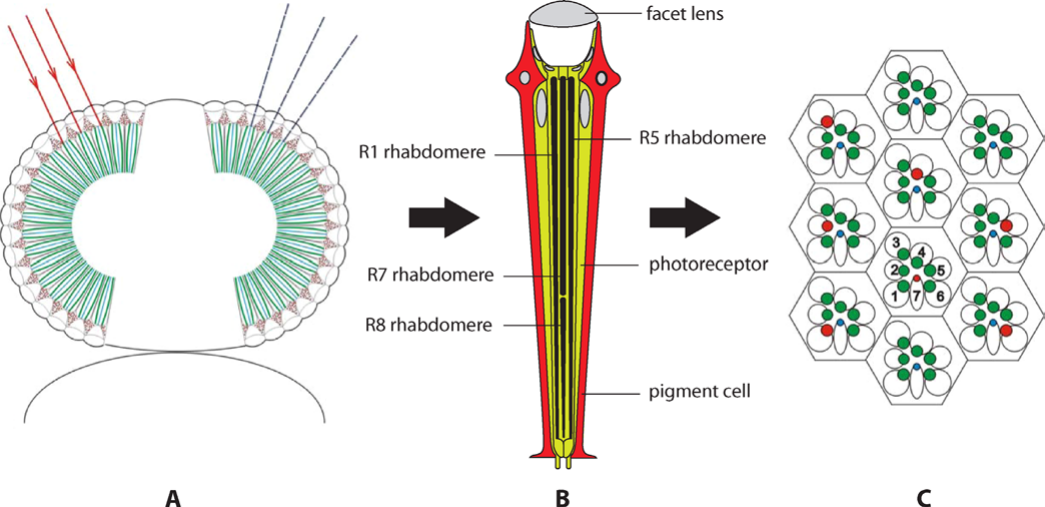
Diagrams of the Eye and Retinal Organization of the Fruit Fly Drosophila (A) The two compound eyes are composed of anatomically identical building blocks, the ommatidia, arranged in a hemispherical shell. The wild-type fruit fly eye is red due to pigment in pigment cells that optically isolate the ommatidia from each other. The angle between the visual axes of adjacent ommatidia (dashed blue lines), about 5°, determines the spatial acuity of the eye. (B) Each fly ommatidium contains eight photoreceptor cells, R1–R8. The six large, outer, or peripheral photoreceptors, R1–R6, have long and fat rhabdomeres, and the two slender, inner, or central photoreceptors, R7 and R8, have thinner rhabdomeres, arranged in tandem, with R7 distal and R8 proximal. (C) Light from a distant point source ((A) red lines with arrows) projected into the R7 of a certain ommatidium is received also by six photoreceptors of the R1–R6 class ((C) red rhabdomeres), each in a different, neighboring ommatidium. The signals of these R1–R6 photoreceptors are summed in the lamina, the optical ganglion adjacent to the retina, thus providing a high-sensitivity motion vision system. The R7s and R8s feed into a color vision system.

Accordingly, the photoreceptors and their rhabdomeres can be classed as outer and inner (or peripheral and central), respectively [2,5], and these two classes serve different visual functions. R1–R6 mediate highly light-sensitive, broad spectral-band motion vision, and R7 and R8 underlie color vision, a capacity that is enabled by special optical and neural organizations [5]. The two classes of photoreceptors can be not only differentiated by the type of vision they mediate, but also by the route via which the visual information is conveyed. The different classes of visual information are collected in two consecutive optical ganglia—namely, the lamina, where information from R1–R6 is collated, and the medulla, where the R7 and R8 signals are transmitted. The specifics regarding further neural processing are unfortunately largely unknown.

Extensive studies on the larger housefly (Musca) and blowfly (Calliphora) have provided considerable insight into the workings of the fruit fly's retinal organization. For example, transmission light microscopy has revealed two types of ommatidia, with the R7/R8 rhabdomeres of randomly distributed ommatidia appearing yellow, due to a blue-absorbing, photostable pigment (presumably zeaxanthin and/or lutein) concentrated in the R7 rhabdomere, and the rhabdomeres in the complementary set of ommatidia appearing pale [7]. The R7 and R8 photoreceptors of these ommatidia types, accordingly called y and p type, appear to have different rhodopsins [5].

Drosophila has six rhodopsins, Rh1–Rh6. The corresponding protein moieties, the opsins, are encoded by the genes *rh1-rh6* [3,5,8]. Depending on the opsin, the rhodopsin absorbs primarily in one of three wavelength ranges—ultraviolet (UV), blue, or green (Figure 2). The exclusively UV-absorbing Rh3 (Figure 2) occurs in the R7 photoreceptors of 30% of the ommatidia, which are randomly distributed throughout the retina. These ommatidia are called p type, based on their similarity to the UV-absorbing rhodopsin in the R7 photoreceptors of housefly and blowfly p type ommatidia. Rh4, which also absorbs UV (Figure 2), is found in the complementary y type ommatidia, which make up the remaining 70% of R7 photoreceptors. The blue-absorbing Rh5 opsin is expressed in all R8s of the p type ommatidia; the green-absorbing Rh6 opsin is expressed in the R8s of the y type (Figure 2). New research adds a twist to this established model of the fruit fly retina, which has prevailed for about a decade [8]. In this issue of *PLoS Biology*, Mazzoni et al. [9] show that the 70% y type ommatidia are nonhomogeneous: 60% express only *rh4*, whereas 10% co-express *rh3* and *rh4*; the latter localize in the dorsal third of the Drosophila eye.

**Figure 2:**
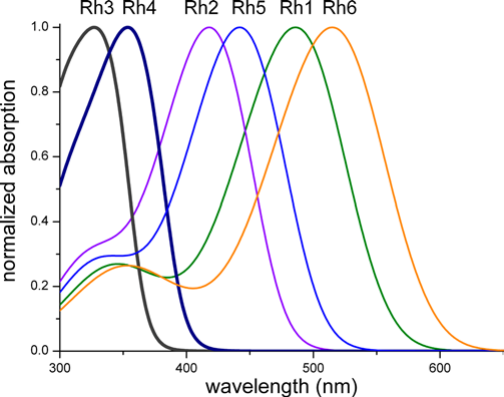
Absorption Spectra of the Six Rhodopsins Expressed by the Fruit Fly Drosophila (after [8]) Rh1 is the rhodopsin of the R1–R6 photoreceptors, Rh2 is expressed in the ocelli, Rh3 and Rh4 exist in two main classes of R7 photoreceptors, and Rh5 and Rh6 are the rhodopsins of the two corresponding classes of R8 photoreceptors. A specific, small set of R7 photoreceptors expresses both Rh3 and Rh4.

A long-standing general principle in vision research holds that single photoreceptors always contain a single type of rhodopsin, although occasional examples of co-expressed rhodopsins, as the authors have now demonstrated for Drosophila, have cropped up in both vertebrates and invertebrates. Röhlich et al. [10], for example, investigated the retinae of the mouse, rabbit, and guinea pig, which are divided into a superior area dominated by green-sensitive (M) cones, and an inferior area in which cones possess practically only short wavelength–absorbing (S) rhodopsins. They found that the transitional zone between these retinal areas is populated by cones labeled by both M and S cone rhodopsin-specific antibodies. A related result was reported for the Syrian hamster [11]. Kitamoto et al. [12] studied the Japanese swallowtail butterfly, Papilio xuthus, and found that the proximal photoreceptors of the so-called type II ommatidia co-express two mRNAs encoding long wavelength–absorbing rhodopsins. Recently, another example of co-expression of rhodopsins, resembling the Drosophila case, was discovered in a restricted area of the compound eye of the Small White butterfly, Pieris rapae crucivora. In a transition area— which is approximately six ommatidia wide—between the dorsal area and the main dorsoventral area, the short-wavelength photoreceptors express two RNAs coding a UV-absorbing and a blue-absorbing rhodopsin (Omiya M and Arikawa K, unpublished data). The spatial area covered by these photoreceptors is similar to those of the co-expressing Drosophila photoreceptors discovered by Mazzoni et al. [9], suggesting that they fulfill a similar visual role (see below).

An obvious question is whether the co-expressed rhodopsins are both functional—that is, whether they are capable of triggering the phototransduction process, thus inducing a visual signal proportional to the absorbed photon flux. That question has been positively answered for the Papilio xuthus photoreceptors that co-express two rhodopsin mRNAs. Intracellular electrophysiological recordings of the spectral sensitivity of these photoreceptors yielded very broadband sensitivity spectra, which could be interpreted with a computational model that assumed additive sensitivity of two phototransduction chains driven by the two rhodopsins [13].

We emphasize here that double expression of two rhodopsins does not produce an enhancement of absolute light sensitivity. Indeed, Mazzoni et al. [9] found that less Rh3 exists in R7 cells that co-express *rh3* and *rh4* than in R7 cells expressing only *rh3*. Assuming that the Rh3 and Rh4 expressed by the Drosophila R7 photoreceptors participate equally in the phototransduction process, the spectral sensitivity of the R7s will be proportional to the summed absorption spectra of the two UV rhodopsins. Because the absorption spectra of Rh3 and Rh4 are not widely spaced apart (Figure 2), the spectral sensitivity of the R7s will only be slightly broadened and still be restricted to the ultraviolet range. (Note that the spectral properties of the ommatidium's integrated optics, consisting of the diffracting facet lens and the waveguiding rhabdomere, also contribute to the spectral sensitivity of a photoreceptor cell, although to a minor extent [14].)

Because double expression of the UV rhodopsins occurs in the R7s in the dorsal third of the compound eye, Mazzoni et al. [9] hypothesize that the R7 photoreceptors, together with the underlying R8s, function in analyzing the UV sky specifically to detect differences in the solar and nonsolar parts of the sky—that is, sky near the sun and away from it—which can differ considerably in short-wavelength light content. This skylight-discriminating ability may serve to help the fly orient for navigational purposes. Although testing this hypothesis will be difficult, because Drosophila is not well known for easy behavioral experiments, calculations of light capture of different sky areas based on detailed modeling of photoreceptor sensitivities will illuminate the tenability of the offered hypothesis. Alternatively, experiments on other species with similar expression patterns, like the butterflies, may provide behavioral evidence.

The exciting, novel thrust coming from the Mazzoni et al. [9] study is their contribution toward unraveling the genetic pathway underlying *rhodopsin* co-expression. Although many details still have to clarified, it is now clear that *spalt* genes control the destiny of the R7 and R8 photoreceptors in the first phase of retinal development. In the dorsal-most row of ommatidia, *homothorax* then governs the expression of the UV-absorbing Rh3 in both R7 and R8, so that these photoreceptors can mediate polarization vision [15,16]. The expression of *orthodenticle* is a necessary step in the generation of Rh3 and Rh5 rhodopsins in the R7 and R8 of p type ommatidia, and *spineless* determines the fate of y type ommatidia, with the Rh4 and Rh6 rhodopsins in the R7 and R8 photoreceptors, respectively.

In their careful analysis, Mazzoni et al. [9] have now demonstrated that co-expression of Rh3 and Rh4 occurs in a restricted number of y type R7 photoreceptors under the command of genes of the Iroquois Complex (Iro-C), a family of genes found in nematodes, insects, and vertebrates [17]. The Iroquois genes, discovered in Drosophila, play a crucial role in neural development, for instance in the mouse and zebrafish retina [18,19]. The first mutant allele recovered in Drosophila suppressed all of the lateral bristles of the dorsal mesothorax (notum), leaving only a wide band of both large and small bristles in the central region of the notum: a pattern resembling the hairstyle of the Iroquois North American Indians (also known as Mohawk) [17]. The Drosophila Iro-C genes encode the transcription factors *araucan* (*ara*; after the Araucanian South American Indians), *caupolican* (*caup*; after Caupolicán, a South American Indian leader), and *mirror* (*mirr*), which have homologs in vertebrates [17,20,21]. Presumably *ara* and *caup* lift the blockade of the expression of Rh3 in the commonly Rh4-expressing y type R7.

It will be of great interest to see whether the dual expression of rhodopsins as found in the fruit fly is under similar genetic control, in butterfly as well as in vertebrate photoreceptors. This may further improve our insight into the evolution of rhodopsin expression patterns. Surveying different Drosophila species using the well-filled genetic toolbox now available for D. melanogaster will undoubtedly be of great value to further uncover the evolutionary history as well as the genetic mechanisms that break the one rhodopsin-per-photoreceptor rule.
